# Case Report: Long-term remission of metastatic pancreatic ductal adenocarcinoma after surgery and high-dose nicotinamide supplementation

**DOI:** 10.3389/fonc.2026.1927317

**Published:** 2026-09-03

**Authors:** Hubert Kolb, Werner Hartwig, Torsten Beyna, Daniela auf der Horst, Kerstin Kempf, Stephan Martin, Kerstin Struse-Soll, Markus Dommach

**Affiliations:** 1 Faculty of Medicine, University of Düsseldorf, Düsseldorf, Germany; 2 West-German Centre of Diabetes and Health, Düsseldorfer Kliniken der Franziskus-Stiftung, Düsseldorf, Germany; 3 Department of Surgery, Evangelisches Krankenhaus Düsseldorf, Düsseldorf, Germany; 4 Department of Internal Medicine, Evangelisches Krankenhaus Düsseldorf, Düsseldorf, Germany; 5 Oncology Medical Care Center, Sana Clinics Gerresheim, Düsseldorf, Germany

**Keywords:** Ca19-9, liver metastases, NAD^+^, nicotinamide, pancreatic ductal adenocarcinoma, poly-ADP-ribose polymerase

## Abstract

**Background:**

Pancreatic ductal adenocarcinoma carries a poor prognosis, with the worst outcomes observed in UICC stage IV disease characterized by hepatic—and less frequently pulmonary—metastases. Considerable genetic heterogeneity, including mutations in *KRAS*, *BRCA1/2*, and *ERBB2*, enables the development of individualized therapeutic strategies. We report here a patient with *KRAS*- and *BRCA1/2*-wild-type but mutant *TP53* and *ERBB2* disease who achieved apparent remission of hepatic metastases.

**Case presentation:**

A 78-year-old Caucasian male presented with newly detected dilation of the distal main pancreatic duct and prepapillary stenosis associated with a hypodiffused region. After 8 months of surveillance, rising CA19-9 levels prompted pylorus-preserving pancreaticoduodenectomy. Two small hepatic nodules were resected and confirmed as metastatic lesions. The primary tumor was identified as invasive micropapillary pancreatic ductal adenocarcinoma (PDAC). Surgery did not slow or halt the progressive rise of CA19–9 levels in serum. Adjuvant gemcitabine therapy was discontinued at the patient’s request after four infusions, and high-dose nicotinamide treatment (3 x 500 mg per day) was initiated 11 weeks later. CA19-9 levels declined from 1376 U/ml to the normal range within 5 months. Initial magnetic resonance imaging revealed multiple small hepatic metastases, which regressed in parallel with CA19-9 levels and were no longer detectable one and two years later.

**Conclusion:**

Regression of CA19-9 levels and imaging-detectable hepatic metastases occurred concurrently after initiation of high-dose nicotinamide. This temporal relationship may be incidental, and the remission could represent a delayed effect of the preceding gemcitabine infusions. Nicotinamide supplementation may also have contributed anti-tumor activity. High-dose nicotinamide elevates systemic NAD^+^ levels, which may enhance immune function within the metastatic tumor microenvironment. In addition, partial inhibition of poly(ADP-ribose) polymerase by high-dose nicotinamide can impair DNA repair in tumor cells with underlying repair deficiencies, thereby promoting tumor cell death.

## 1 Introduction

Although pancreatic cancer accounts for only 2–3% of all cancers reported in global GLOBOCAN and European ECIS databases, its mortality burden is disproportionately high, ranking between 4.8% and 8% (1, 2). At diagnosis, more than half of patients present with metastatic disease, most commonly to the liver (3). Median survival for unselected patients with pancreatic ductal adenocarcinoma (PDAC) and liver metastases is 4–7 months, compared with 8–12 months in those undergoing cancer directed surgery (4). A recent systematic review of oligometastatic PDAC reported overall median survival of 6–9.9 months without surgery versus 7.6–18.4 months in patients who underwent pancreatic tumor resection (5). We describe here an unexpected case of long-term remission in a patient with oligometastatic PDAC.

## 2 Case presentation

A 78-year-old Caucasian male presented with a newly detected dilation of the distal main pancreatic duct (4 mm) on routine abdominal ultrasound during an annual health check, not observed in previous years. Blood tests indicated an otherwise healthy status, with stable mild leukopenia and erythropenia (**Table 1**). Magnetic resonance cholangiopancreatography (MRCP) and computed tomography revealed prepapillary stenosis and prestenotic ductal dilation up to 6 mm. No definitive malignant mass was identified, although a suspicious region measuring up to 1 cm was noted and subsequently confirmed by endoscopic ultrasonography. Resection of the pancreatic head was recommended, but the patient opted for continued surveillance. Three follow-up MRCPs and two endoscopic ultrasonographies showed no obvious progression. The previously noted suspicious area appeared as a mildly hypodiffuse 7-mm region without measurable growth. An endoscopic biopsy at month 8 yielded no malignant cells.

**Table 1:** Follow-up of basic patient data.

		Time from tentative diagnosis			
Parameters^a^		-12 months	0^b^ months	+15 months^c^	+40 months
Body weight (kg)	61		59	56	57
Blood glucose (mg/dl)	77		81	83	92
GGT (U/l)		15	32	111	25
AP (U/l)		56	73	216	107
Hemoglobin (g/dl)		12.7	13.4	9.8	11.1
Hematocrit (%)		37.7	40.3	30.5	32.7
Leukocytes (per µl)		2560	2730	2980	3380
Lymphocytes (%)		37.9	37.0	22.8	21.6
Neutrophils (%)		39.7	42.2	61.8	58.2
Thrombocytes (per nl)		221	272	334	210

^a^All parameters determined pre-lunch.

^b^Pancreas head resection at 10 months, chemotherapy during month 12.

^c^Start of nicotinamide supplementation at 15 months.

GGT, Gamma-glutamyl-transferase; AP, alkaline phosphatase.

**Figure 1:**
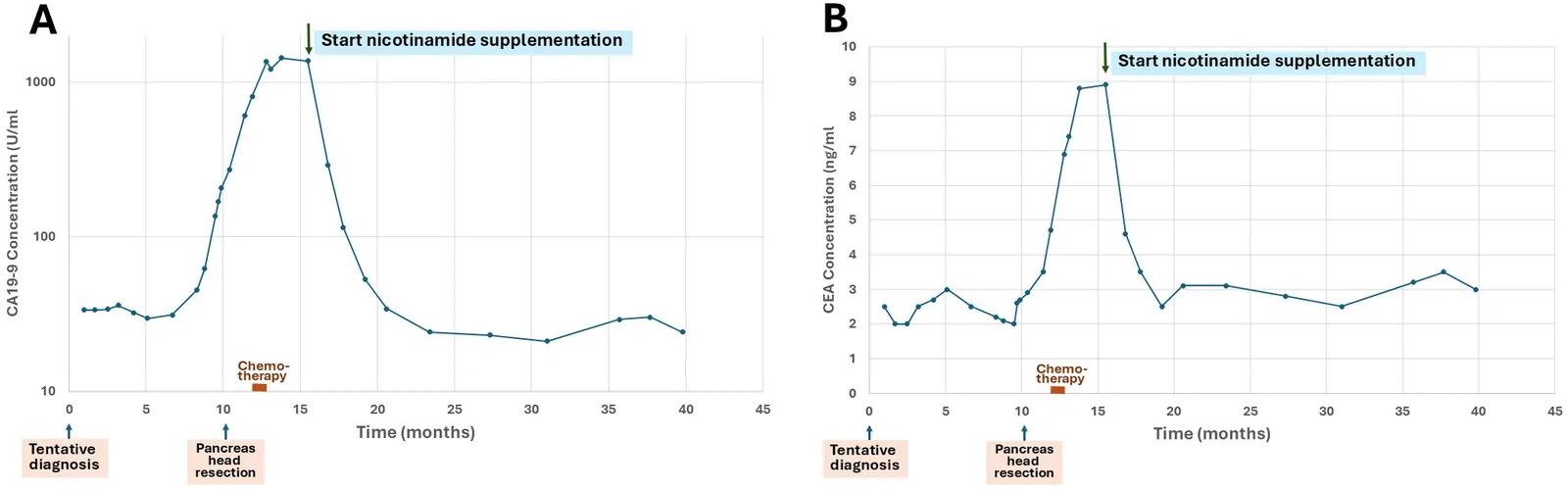
Course of tumor-marker concentrations in serum in relation to interventions. **(A)** Trajectory of CA19-9 levels (upper normal range limit <34 U/ml). **(B)** Trajectory of CEA levels (upper normal range limit <3.0 ng/ml). Chemotherapy consisted of four infusions administered during month 12. Daily nicotinamide supplementation began at 15.4 months and was continued without interruption.

A continuous rise in serum CA19-9 levels was observed from month 8 onward, prompting pylorus-preserving pancreaticoduodenectomy at month 10 (**Figure 1A**). Intraoperatively, two small liver lesions were detected and resected. Histopathology confirmed both as metastatic pancreatic ductal adenocarcinoma, measuring 10 mm and 3 mm, respectively. In the pancreatoduodenectomy specimen, histopathology revealed a 1.6-cm invasive micropapillary pancreatic adenocarcinoma with metastases in 5 of 22 examined lymph nodes, tumor grade 3, [pT1c pN2 (5/22, ECE+) L1 V1 Pn1 G3 pM1(hep) R0 (CRM+ dorsal)], UICC stage IV.

**Figure 2:**
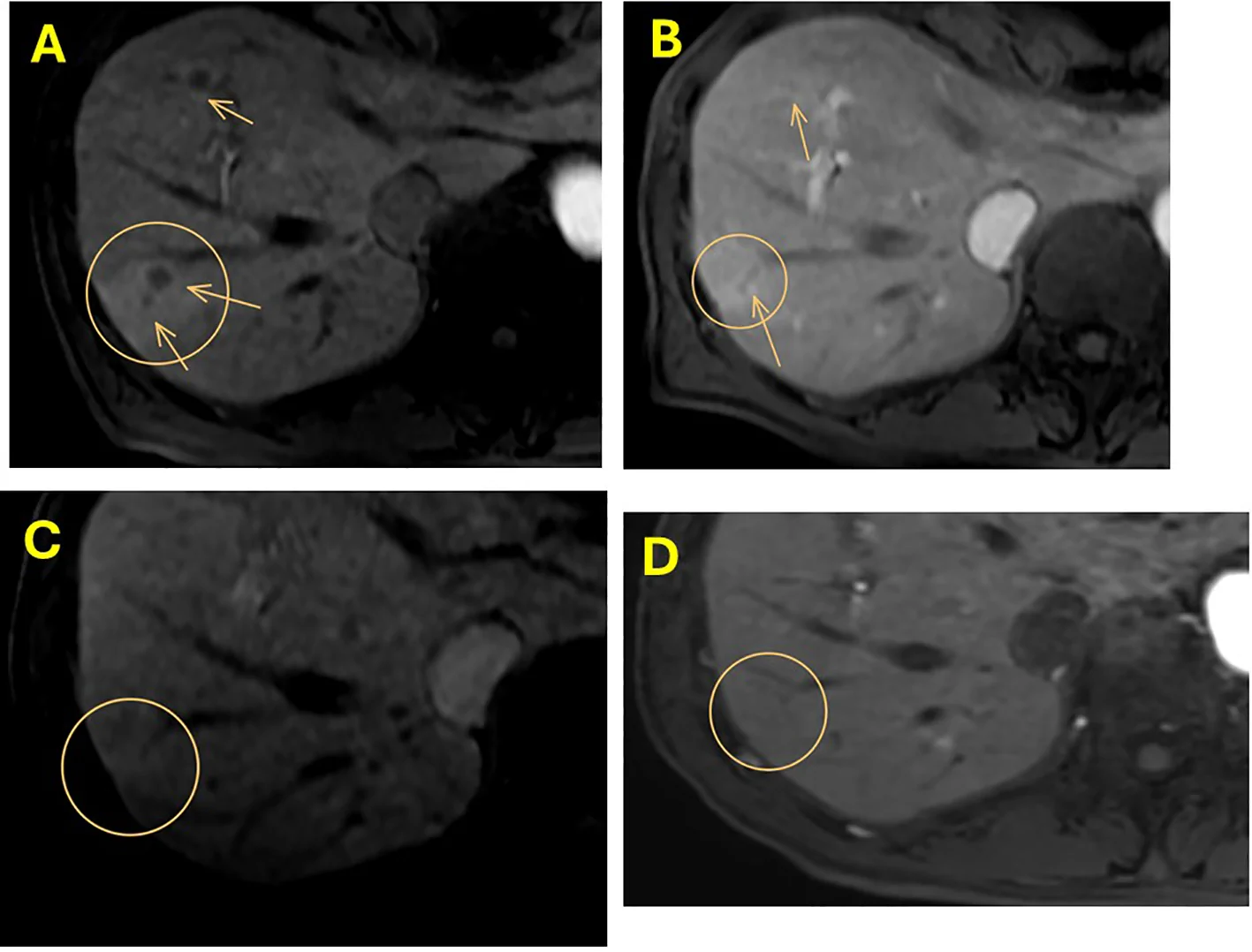
**(A)** Seven weeks post-surgery, MRCP revealed several small metastatic lesions (see arrows) distributed throughout the liver parenchyma. **(B)** After 5 months of nicotinamide therapy, follow-up MRCP demonstrated marked regression (see arrows) or disappearance of these lesions (same position of the circle as before). **(C)** A subsequent scan 3 months later confirmed complete radiological resolution of metastatic disease (same position of the circle as before). **(D)** At the end of the observation period (41 months after tentative diagnosis, 31 months after surgery, 25 months after initiation of nicotinamide), MRCP again showed no detectable metastases (same position of the circle as before). Two elongated lesions measuring 6 mm and 17 mm remained stable for over 2 years and likely represent residual fibrotic tissue (not depicted here). All four panels depict the same liver section, as indicated by the identical arrangement and trajectory of the liver venules.

At hospital discharge, serum CA19-9 levels were 270 U/ml and continued to rise, paralleled by increasing CEA concentrations (**Figure 1**). MRCP performed seven weeks after surgery revealed several new small hepatic metastases (**Figure 2A**). Owing to the patient’s advanced age and frail condition, monotherapy with gemcitabine (1.69 g) was initiated eight weeks postoperatively. After the second weekly infusion, neutrophil counts declined markedly, prompting a 50% dose reduction for the third infusion. The second treatment cycle began after a two-week break but was discontinued after the first session because of nausea, reduced physical fitness, and persistently rising CA19-9 levels, which indicated ongoing progression of hepatic metastases. The patient elected to stop further chemotherapy.

After partial recovery, therapeutic options were reconsidered, and high-dose nicotinamide supplementation (3 × 500 mg/day) was initiated 11 weeks later based on encouraging reports in skin cancer, ongoing trials in other tumor types, and a plausible molecular rationale (see Discussion). The discussion of potential treatment options and the decision to initiate nicotinamide therapy were undertaken with active patient involvement. Serum levels of CA19-9 plateaued for 11 weeks after chemotherapy was discontinued. Following the start of nicotinamide, serum CA19-9 levels declined rapidly—by 79% within five weeks—and normalized within five months. Elevated liver enzymes gamma-glutamyl-transpeptidase (GGT) and alkaline phosphatase (AP) also decreased (**Table 1**), returning to the normal range within 10 weeks. During the current two-year follow-up period after initiation of nicotinamide, CA19-9 levels have remained within the normal range (**Figure 1A**), and CEA levels showed a parallel trajectory (**Figure 1B**). Analyses by computed tomography and MRCP at 5 months of nicotinamide supplementation did not show metastases outside the liver and demonstrated continued regression or disappearance of imaging-detectable hepatic metastases and no evidence of new lesions (**Figure 2B**). Two further analyses by MRCP confirmed disappearance of imaging-detectable hepatic metastases and no regrowth until the end of the observation period (**Figures 2C, D**). At the times when tumor markers were measured, complete blood work was also performed. No detrimental effects of high-dose nicotinamide on blood cells or on liver or kidney function parameters were observed.

Genomic analysis of the primary tumor revealed an uncommon pattern of wild-type and mutated genes. Sequencing of more than 500 tumor-associated genes identified pathogenic mutations in *ERBB2* and *TP53*, while *KRAS*, *BRCA1/2*, and *CDKN2A/B* remained wild-type. Two genes involved in chromatin remodeling, *EP300* and *KMT2D* were mutated but these were variants of unknown significance. RNA-based screening for gene fusion events across 49 cancer-associated genes detected no hybrid transcripts. Immunohistochemical assessment of 10 potentially overexpressed oncogenic proteins showed strong staining for human epidermal growth factor 2 (HER2), the ERBB2 gene product (immunohistochemistry grade 2+). This finding was corroborated by silver *in situ* hybridization, which demonstrated marked ERBB2 gene amplification with an average of 12.6 copies per cell (positivity defined as ≥4 copies per cell).

## 3 Discussion

In the case presented, tumor growth appeared to follow two distinct phases. At the time of tentative diagnosis, the lesion presented as a small hypodiffuse area measuring 0.7 cm in diameter. Serial imaging showed no enlargement for many months, and serum CA19-9 concentrations remained low and stable within the upper normal range. Approximately two months before surgery, CA19-9 levels began to rise, likely in parallel with hepatic metastasis, as suggested by intraoperative detection of two liver lesions (3 mm and 10 mm), which were resected together with the primary tumor (**Figure 1A**). The absence of a postoperative decline in CA19-9, but a continued steady increase, indicated that metastatic tumor cells were the major source of excessive CA19-9 production. Because the two visible liver metastases had been removed, the most likely source of circulating CA19-9 was the numerous small metastases detectable only by MRCP (**Figure 2A**). These observations suggested a transition from a slowly growing, CA19-9–negative tumor phenotype to a rapidly proliferating, CA19-9–positive phenotype.

The latter tumor phenotype was likely nonresponsive to gemcitabine. Serum CA19-9 increased from 810 U/ml before treatment to 1434 U/ml after discontinuation of gemcitabine, and CEA rose concurrently from 4.7 to 8.8 ng/ml. Chemotherapy was stopped after the first infusion of cycle 2. A favorable response to gemcitabine is typically associated with a >20 % decrease in CA19-9 after two cycles, whereas after one cycle the maximal increase compatible with improved outcome is <5 % (6). Given the observed 67 % rise after one cycle, a subsequent decline below baseline after the second cycle was highly unlikely. Although disappointing, this pattern is consistent with the reported response rate of <10 % for patients with advanced pancreatic cancer receiving standard gemcitabine therapy (7). Still, it should be noted that the serum levels of tumor markers CA19–9 and CEA reached a plateau for 2–3 months after chemotherapy which may be a consequence of the short period of gemcitabine treatment.

As an aggressive solid tumor, pancreatic ductal adenocarcinoma and its liver or lung metastases respond poorly to current chemotherapeutic regimens, although combination therapies have achieved modest improvements in progression-free survival (8). The metastatic tumor microenvironment is characterized by a severe immunosuppressive milieu, which likely contributes to the limited efficacy of immunotherapies, including immune checkpoint inhibitors and CAR T-cell approaches targeting individual or shared tumor antigens (9–13). A major driver of this immunosuppressive state appears to be NAD^+^ deficiency, potentially caused by NADase activity—such as CD38 expressed on immune and non-immune stromal cells—and by depletion through highly NAD^+^-dependent tumor cells (14–16).

Increasing systemic NAD^+^ levels—whether by direct infusion or by administering precursors such as nicotinamide—represents a double-edged strategy. While elevated NAD^+^ may counteract the immunosuppressive milieu of the metastatic tumor microenvironment, it can also raise intracellular NAD^+^ in tumor cells and potentially promote tumor growth (16, 17). Indeed, several therapeutic approaches aim to inhibit tumor growth by suppressing NAD^+^ availability (18, 19). Despite this theoretical risk, clinical trials using high-dose nicotinamide (typically 0.5–1.5 g/day) have been conducted in individuals with malignancies or at elevated cancer risk. To date, nicotinamide-induced tumor progression has not been reported. Most studies have focused on patients with skin cancer or those at high risk. Although many trials report reduced incidence of skin cancer or actinic keratosis—and decreased progression of existing lesions—a definitive consensus remains lacking (20–23). The ClinicalTrials.gov database lists nearly 700 interventional studies investigating nicotinamide or nicotinamide riboside for the prevention or treatment of various cancer types (accessed July 27, 2026). No clinical trials have been reported in patients with pancreatic cancer at any stage.

A second pharmacological action of nicotinamide is the inhibition of poly(ADP-ribose) polymerases (PARP) and, to a lesser extent, mono-ADP-ribose transferases (24). *In vivo* studies report a half maximal inhibitory concentration (IC_50_) of approximately 0.1 mmol/L for PARP inhibition by nicotinamide (24, 25). The high nicotinamide dose administered in the present case is expected to reach circulating concentrations of 0.1–0.3 mmol/L, sufficient to induce substantial PARP inhibition. We previously demonstrated that PARP activation in pancreatic β-cells under inflammatory or chemical stress leads to NAD^+^ depletion and insulin-deficient diabetes, which can be prevented by pharmacological or genetic PARP inhibition (24, 26). Nicotinamide supplementation may therefore help preserve NAD^+^ levels within the tumor microenvironment.

Poly(ADP-ribosyl)ation is a post-translational protein modification involved in the regulation of gene expression (27). Among the 17 PARP family members, PARP1 is the most extensively studied; it is activated by DNA damage and plays a central role in base excision repair. In tumor cells with defective homologous recombination, inhibition of PARP by targeting its nicotinamide binding pocket prevents the repair of single-strand breaks, leading to their conversion into double-strand breaks and ultimately cell death. In contrast, organisms with otherwise intact DNA repair pathways tolerate pharmacological PARP suppression well. High-dose nicotinamide has shown no apparent adverse effects, consistent with findings from large placebo-controlled trials conducted over several years, some of which included children (20, 28, 29). PARP inhibitors such as olaparib and further nicotinamide mimics have been successfully incorporated into the treatment of cancers with known or suspected defects in DNA repair (30, 31). During olaparib therapy, plasma concentrations typically exceed the IC_50_ by a factor of 10–50, whereas gram-level nicotinamide dosing achieves only IC_50_-range concentrations (32, 33). Another key distinction is that most pharmacological PARP inhibitors are not NAD^+^ precursors; consequently, the physiological effects of high-dose nicotinamide supplementation cannot be directly equated with those of PARP inhibitors, although some mechanistic overlap is expected.

Although a plausible molecular mechanism exists for remission induction by high-dose nicotinamide, a single case cannot establish causality. The temporal association between initiation of nicotinamide supplementation and the decline in CA19-9 levels may be coincidental. We did not assess whether high-dose nicotinamide treatment in the patient affected NAD^+^ levels or PARP activity. Regression of CA19-9 and CEA serum levels, along with the reduction of imaging-detectable hepatic metastases, may represent a delayed response to the short course of gemcitabine.

In this patient, genomic analysis showed wild-type *BRCA1* and *BRCA2*; however, variants in other genes or gene-expression programs could have impaired homologous recombination or other DNA-repair pathways causing susceptibility to PARP inhibition. Regardless of the underlying mechanism, the rapid and durable decline in CA19-9 and CEA levels, together with remission of hepatic metastases despite minimal chemotherapy, is remarkable. This response may be linked to the patient’s atypical genetic profile, characterized by absence of *KRAS* mutations but presence of *TP53* and *ERBB2* oncogenic alterations, although both are associated with poor prognosis (34, 35).

Pancreatic resection or biopsy specimens lack detectable *KRAS* mutations in 9–12% of cases, and in 7–8% of advanced cases (36–39). There is substantial heterogeneity in other potentially oncogenic mutations, along with a more than fourfold increase in overall mutational burden, making classification by genetic profile in relation to prognosis challenging. For example, *ERBB2* mutation and overexpression—as observed in the present case—occur in only about 2% of patients with *KRAS* wild-type tumors. *TP53* is the most frequently mutated gene, reaching approximately 80% in cases of oligometastatic PDAC. Epigenetic alterations add an additional layer of heterogeneity to pancreatic cancer. Although it is widely believed that patients with *KRAS* wild-type tumors have prolonged survival, the small number of such cases—particularly within the subgroup presenting with oligometastatic PDAC at the time of resection—precludes firm conclusions (36–40).

How does the present case compare with previously reported instances of unexpected long-term remission in oligometastatic pancreatic cancer? Population-based data from the U.S. National Cancer Institute indicate that cancer-directed surgery confers a modest survival benefit. Among patients older than 70 years at diagnosis, surgical intervention increased median survival from 4 to 6 months. In patients younger than 60 years, median survival rose from 8 to 12 months following pancreatic resection (4). These figures underscore that durable remission of hepatic metastases and multi-year survival without relapse lie far outside established statistical expectations. Two cases of apparently complete, multi-year remission have been described following partial pancreatic resection, adjuvant chemotherapy, and radiofrequency ablation of liver metastases. However, no information on the molecular or genetic profiles of the tumor specimens was provided in either report (41, 42). In another case, a 63-year-old woman developed hepatic metastases despite adjuvant chemotherapy after pancreaticoduodenectomy. Following 15 cycles of gemcitabine, the liver lesions regressed completely, and no recurrence was observed for more than seven years. As with the previous reports, molecular characterization of the primary tumor was not available (43).

Two patients with tumors located in the pancreatic head were found to carry *BRCA2* mutations in tumor biopsies and achieved apparently complete remission of hepatic metastases after platinum-based chemotherapy, without undergoing cancer-directed surgery. Both remained disease-free for more than six years. *KRAS* status was not reported in either case (44).

Taken together, these observations suggest that rare instances of long-term, apparently complete remission can occur both in patients who undergo cancer-directed surgery and in those treated exclusively with systemic therapy. Notably, remission in all reported cases emerged in the context of prolonged chemotherapy. We did not identify any case comparable to the present one—namely, long-term remission following surgery and only 1.3 cycles of gemcitabine. The potential contribution of high-dose nicotinamide remains speculative.

## Data Availability

The raw data supporting the conclusions of this article will be made available by the authors, without undue reservation.
